# Selective Inhibitors of the Inducible Nitric Oxide Synthase as Modulators of Cell Responses in LPS-Stimulated Human Monocytes

**DOI:** 10.3390/molecules26154419

**Published:** 2021-07-22

**Authors:** Marialucia Gallorini, Monica Rapino, Helmut Schweikl, Amelia Cataldi, Rosa Amoroso, Cristina Maccallini

**Affiliations:** 1 Department of Pharmacy, University “G. d′Annunzio” Chieti-Pescara, Via dei Vestini 31, 66100 Chieti, Italy; marialucia.gallorini@unich.it (M.G.); cataldi@unich.it (A.C.); cristina.maccallini@unich.it (C.M.); 2 Genetic Molecular Institute of CNR, Unit of Chieti, “G. d′ Annunzio” University, Via dei Vestini 31, 66100 Chieti, Italy; monica.rapino@unich.it; 3 Department of Conservative Dentistry and Periodontology, University Hospital Regensburg, D-93042 Regensburg, Germany; helmut.schweikl@klinik.uni-regensburg.de

**Keywords:** immunomodulation, inflammation, inhibitors, monocytes, nitric oxide

## Abstract

Inducible nitric oxide synthase (iNOS) is a crucial enzyme involved in monocyte cell response towards inflammation, and it is responsible for the production of sustained amounts of nitric oxide. This free radical molecule is involved in the defense against pathogens; nevertheless, its continuous and dysregulated production contributes to the development of several pathological conditions, including inflammatory and autoimmune diseases. In the present study, we investigated the effects of two new iNOS inhibitors, i.e., 4-(ethanimidoylamino)-*N*-(4-fluorophenyl)benzamide hydrobromide (FAB1020) and *N*-{3-[(ethanimidoylamino)methyl]benzyl}-l-prolinamidedihydrochloride (CM554), on human LPS-stimulated monocytes, using the 1400 W compound as a comparison. Our results show that CM544 and FAB1020 are selective and decrease cytotoxicity, IL-6 secretion and LPS-stimulated monocyte migration. Furthermore, the modulation of iNOS, nitrotyrosine and Nrf2 were analyzed at the protein level. Based on the collected preliminary results, the promising therapeutic value of the investigated compounds emerges, as they appear able to modulate the pro-inflammatory LPS-stimulated response in the low micromolar range in human monocytes.

## 1. Introduction

Monocytes are a phenotypically heterogeneous pool of immune cells acting as precursors of macrophages, dendritic cells, and osteoclasts. They also act as first-line defense cells, secreting pro-inflammatory cytokines and chemokines such as TNF α, IL-1β and CXCL10, and they are involved in the production of reactive oxygen and nitrogen species (ROS and RNS) [1]. In addition to their physiological function as innate effector cells, they also display a central role in the progression of inflammation in different diseases, such as autoimmune diseases, cancer, and atherosclerosis [2,3,4].

During inflammation, monocytes have been shown to preferentially differentiate into inflammatory macrophages in response to local macrophage colony-stimulating factor (M-CSF), a process triggered by physiological stress and inflammatory cues [5]. Moreover, the angiogenic and chemotactic activity of human monocytes also requires the involvement of nitric oxide synthase (NOS) [6]. This is a family of three isoenzymes including the constitutively expressed neuronal NOS (nNOS) and endothelial NOS (eNOS), and the inducible iNOS. All the three isoforms catalyze the conversion of the natural substrate L-arginine into L-citrulline and nitric oxide (NO). This free radical molecule is produced by several types of cells and has many physiological functions including host innate immunity [7]. In fact, the NO produced in macrophages and other innate immune cells is pro-inflammatory and has an essential role in the host immune response against various pathogens, including bacteria, parasites, and viruses. It reacts with the superoxide anion (O_2_^−^) to generate the oxidant peroxynitrite (ONOO^−^), and both NO and ONOO^−^ exert their biocidal effect by targeting many microbial biomolecules leading to protein modifications and DNA damage [8]. Under pro-inflammatory conditions, monocytes produce NO by means of iNOS, which is responsible for the generation of high NO levels. In general, iNOS is expressed also in inflamed tissues as part of the physiological inflammatory response. However, in different inflammatory diseases, including cancer, there is a dysregulation in the expression of iNOS, with a prolonged over-production of NO sustaining the progression of cell damage [9,10,11]. Therefore, the selective inhibition of the iNOS is considered a potential therapeutic target for those pathological conditions in which this enzyme is dysregulated, and several iNOS inhibitors have been reported to date [12,13]. However, no iNOS inhibitor passed clinical trials, mainly due to poor isoform and tissue selectivity.

**Figure 1:**
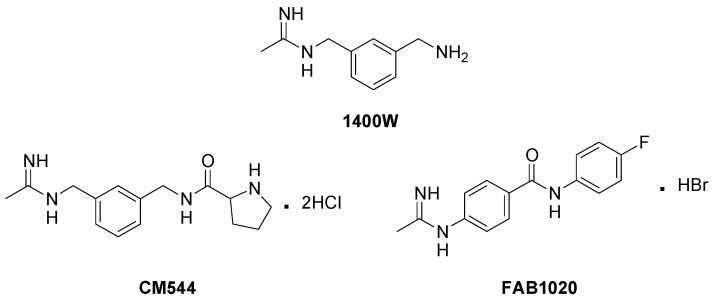
Chemical structure of 1400 W, CM544 and FAB1020.

As part of an ongoing project on the development of safer and potent iNOS inhibitors, we recently reported the compounds *N*-(3-{[(1-iminioethyl)amino]methyl}benzyl)prolinamide dihydrochloride (CM554, Figure 1) and 4-(ethanimidoylamino)-*N*-(4-fluorophenyl)benzamide hydrobromide (FAB1020, Figure 1) [14,15,16]. These acetamidines were designed based on the chemical structure of *N*-(3-(aminomethyl)benzyl)acetamidine (1400 W, Figure 1), a very potent and selective iNOS inhibitor patented by GlaxoSmithKline [17] which, unfortunately, never passed clinical trials. Importantly, these compounds show an excellent degree of selectivity with respect to eNOS, whose activity is essential to maintain the cardiovasculature homeostasis. Previously reported docking studies [14] show that CM544 interactions within the iNOS binding site are very similar to those made by 1400 W and in line with those observed for other structurally related compounds [18,19]. The acetamidine moiety interacts by means of a bidentate hydrogen bond with GLU and TRP residues of the iNOS binding site, while the aromatic ring is positioned atop one of the pyrrole rings of the enzyme heme. Finally, the amide N-H group makes a hydrogen bond with one of the two heme propionate arms, and the proline amino group forms a further hydrogen bond with the remaining one (Figure S2). This last interaction is not observed when CM544 is docked into the eNOS binding site, and this could explain the isoform selectivity. Very interestingly, CM544 and FAB1020 demonstrated promising activities as antiglioma agents, compromising cell cycle progression in C6 rat glioma cells without affecting astrocytes, and exhibiting an improved biological profile with respect to 1400 W. CM544 was also able to counteract inflammation in BV2 microglia cells [20].

Based on the important role of NO in monocytes function, migration and differentiation, this work was aimed at evaluating the effects of CM554 and FAB1020 on a circulating monocytes cell line, to investigate whether the iNOS inhibition by these compounds could affect monocytes’ responses to pro-inflammatory stimuli. Indeed, monocyte modulation could be potentially useful in managing those pathological conditions in which they are excessively recruited or functional.

Firstly, compounds effects on metabolic activity of non-stimulated monocytes were evaluated and compared to those obtained on primary cells from the dental pulp (HDPCs), since they are not able to express iNOS physiologically, but only under inflammatory conditions [21]. Then, a pro-inflammatory cell model was established by stimulating monocytes in the presence of LPS. Indeed, LPS is a potent activator of monocytes/macrophages, and is conventionally used to study inflammation, being able to induce in these cells the production of cytokines, such as TNFα, IL1β, IL6, IL8, IL10, IL12, IL15, and TGFβ [22,23]. Then, stimulated monocytes were treated with CM544 and FAB1020 and their effects on cell viability, cytotoxicity occurrence, IL-6 release, cells migration, nitro-tyrosine detection and Nrf-2 expression were evaluated in comparison with the parent compound 1400 W. Here, we present the results of such investigations.

## 2. Results and Discussion

### 2.1. Cell Metabolic Activity and Selectivity of Compounds

**Figure 2:**
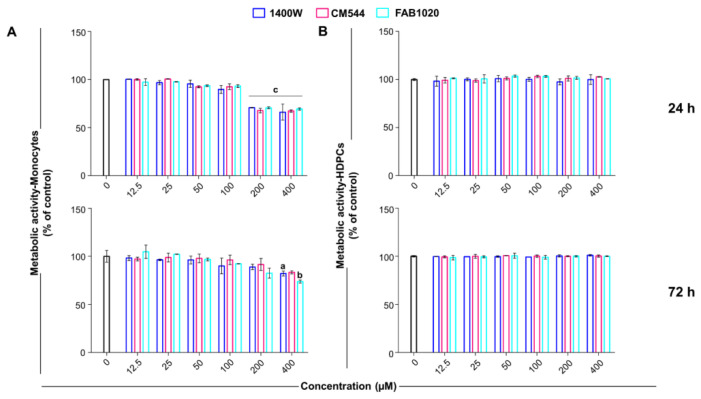
Cell metabolic activity of human monocytes and HDPCs (MTS assay). Cell cultures were exposed to 1400 W, CM544 or FAB1020 for 24 or 72 h. Bars represent the percentages of cell metabolic activity in the presence of compounds (0–400 µM) in human monocytes (**A**) or HPDCs (**B**). OD values in control untreated cultures (0 µM, black bars) are set to 100%. a = *p* < 0.01, b = *p* < 0.001 and c = *p* < 0.0001 between treated cells and the untreated control.

The effect of CM544 and FAB1020 (0–400 µM) was assessed by the MTS assay on human monocytes (Figure 2A) and on HDPCs in parallel (Figure 2B). HDPCs were used here for comparison to demonstrate the selectivity of the iNOS inhibitors using a cell line where this enzyme is not physiologically expressed. Indeed, it was reported that iNOS is expressed in human pulp only in inflamed environments such as pulpitis [18], whereas the iNOS is continuously expressed at low levels in human peripheral blood monocytes [24].

Our results highlight that both CM544 and FAB1020 decrease monocyte metabolic activity only at the highest concentrations tested (200 and 400 µM) to 70% after 24 h, and the same trend is reported for 1400 W (Figure 2). Contrariwise, all compounds are ineffective on HDPCs in the same concentration range after 24 h. After 72 h of exposure, only a concentration of 400 µM seems to be weakly effective on human monocytes, while all compounds are still ineffective on pulp cells.

### 2.2. Cell Metabolic Activity in LPS-Stimulated Monocytes

**Figure 3:**
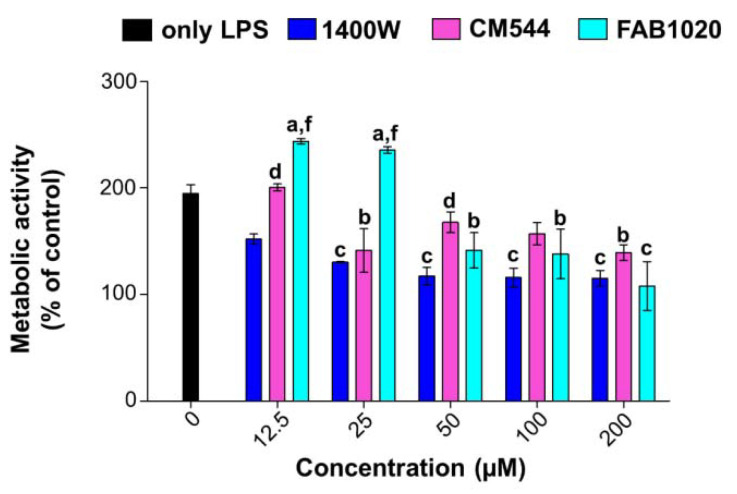
Cell metabolic activity of LPS-stimulated human monocytes (MTS assay). Cell cultures were exposed to 1400 W, CM544 or FAB1020 for 24 h. Bars represent the percentages of cell metabolic activity in the presence of compounds (0–200 µM) in human monocytes stimulated with 0.25 µg/mL of LPS. OD values in control cultures (untreated cells, not shown) are set to 100%. 0 µM = cells stimulated with LPS alone. a = *p* < 0.01, b = *p* < 0.001 and c = *p* < 0.0001 between LPS-stimulated cells and treated with compounds and LPS-stimulated cells (0 µM); d = *p* < 0.01 and f = *p* < 0.0001 between LPS-stimulated cells treated with 1400 W, CM544 or FAB1020 at the same concentration.

After having tested all the compounds in both the cell types to demonstrate selectivity, the biological evaluation was continued only in the presence of human monocytes. In order to induce iNOS protein expression, monocyte cultures were stimulated with 0.25 µg/mL LPS. The LPS concentration able to induce a cell response in human monocytes without being extremely toxic was chosen according to preliminary experiments shown in Figure S1. Indeed, LPS 0.5 µg/mL reduced monocyte metabolic activity by around 80%. Compounds 1400 W, CM544 or FAB1020 were administered in parallel for 24 h up to 200 µM because of the slight cytotoxicity shown for all inhibitors at 400 µM (Figure 2A). The analysis of cell metabolic activity highlights the differential behavior of the two synthetized inhibitors and of 1400 W (Figure 3). Firstly, a stimulation with LPS alone almost doubles (194.8%) the percentage of actively metabolizing monocytes, as a sign of activation [25]. CM544 decreases the metabolic activity of monocytes with respect to LPS alone in a dose-dependent manner starting from 25 µM (141.2%) up to 200 µM (139.4%). As for FAB1020, the percentage of metabolizing cells is clearly increased at lower doses, whereas a fall is registered starting from 50 µM (141.4%). In parallel, 1400 W dramatically decreases the viability of human monocytes already in the lower concentration range (151.9% at 12.5 µM), and this effect is maintained over the doses. Being extremely comparable to 100 µM as regards cell metabolic activity, testing of the 50 and 200 µM concentrations was excluded in further experiments.

### 2.3. Cytotoxicity Occurrence

**Figure 4:**
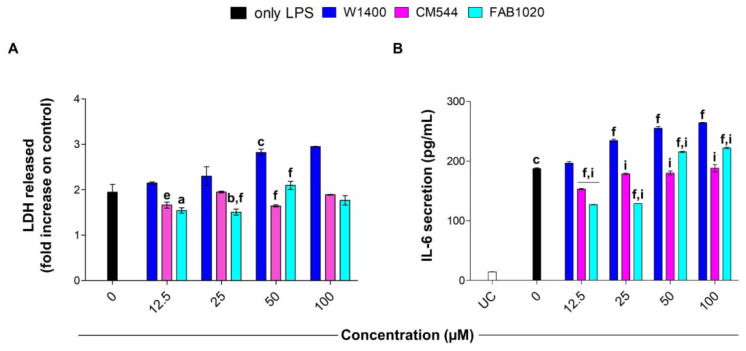
Cytotoxicity and interleukin-6 secretion in LPS-stimulated human monocytes in the presence of increasing concentrations of 1400 W, CM544 and FAB1020 after 24 h. (**A**) Bars represent the amount of LDH released in the presence of compounds (0–100 µM) as the fold increase compared to control samples (untreated cells, not shown) is set to 1. a = *p* < 0.01, b = *p* < 0.001 and c = *p* < 0.0001 between LPS-stimulated cells and LPS-stimulated cells treated with compounds. e = *p* < 0.001 and f = *p* < 0.0001 between LPS-stimulated cells treated with 1400 W, CM544 or FAB1020 at the same concentration. (**B**) Bars show the amount of IL-6 released in the same condition of the LDH assay. UC = untreated cells. c = *p* < 0.0001 between LPS-stimulated cells and untreated cells; f = *p* < 0.0001 between LPS-stimulated cells and LPS-stimulated cells treated with compounds; i = *p* < 0.0001 between LPS-stimulated cells treated with 1400 W and LPS-stimulated cells treated with CM544 and FAB1020 at the same concentration.

To evaluate whether the compounds are capable of counteracting the LPS-induced monocyte activation and cytotoxicity, the release of LDH from human monocytes was quantified (Figure 4A). Stimulation of cells with 0.25 µg/mL LPS increases LDH release about 2-fold compared to untreated cultures (set to 1, not shown). Increasing concentrations of 1400 W are cytotoxic, as revealed by the increased amount of LDH released from monocytes (around 2.9 fold at 50 and 100 µM). Contrariwise, cells in the presence of CM544 or FAB1020 release less LDH compared to monocytes in the presence of LPS alone. For instance, the LDH amount is around 1.5 times higher with regard to untreated cultures, already at 12.5 µM for both compounds.

### 2.4. IL-6 Secretion

IL-6 is a pleiotropic cytokine which plays an important role in host defense against pathogenic microorganisms. During infection, it is promptly produced by monocytes, contributing to the removal of infectious agents and the restoration of damaged tissues through the activation of immune, hematological, and acute-phase responses. Persistent IL-6 production plays a pathological role in the development of various inflammatory diseases and cancers, indicating that IL-6 is a double-edged sword for the host [26]. Moreover, it has been well established that nitrosative and inflammatory stress in LPS-treated cells are induced via TLR4/NF-κB-mediated iNOS expression and IL-6 secretion in LPS-stimulated immune cells [27]. In this light, the amount of IL-6 was quantified in LPS-stimulated monocytes in the presence of iNOS inhibitors after 24 h (Figure 4B). LPS dramatically induces IL-6 secretion from monocytes (187.7 pg/mL) with respect to untreated cells (14.4 pg/mL). Increasing concentrations of 1400 W fail in reducing the amount of IL-6 released, being even more increased with respect to LPS alone (264.3 pg/mL at 100 µM). On the contrary, IL-6 release from LPS-stimulated cell cultures is slightly but significantly decreased in the presence of CM544 low concentrations (153.3 pg/mL). Moreover, the amount of IL-6 released is maintained at lower levels than that in the presence of the LPS alone up to CM544 100 µM. Compared to CM544, FAB1020 is more effective in decreasing the IL-6 amount in the lowest concentration range (around 128 pg/mL at 12.5 and 25 µM). After these concentrations, the secretion of IL-6 is comparable with that of LPS alone-stimulated cells, but is still lower than that in the presence of 1400 W.

### 2.5. Counteraction of LPS-Stimulated Monocyte Migration in the Presence of iNOS Inhibitors

**Figure 5:**
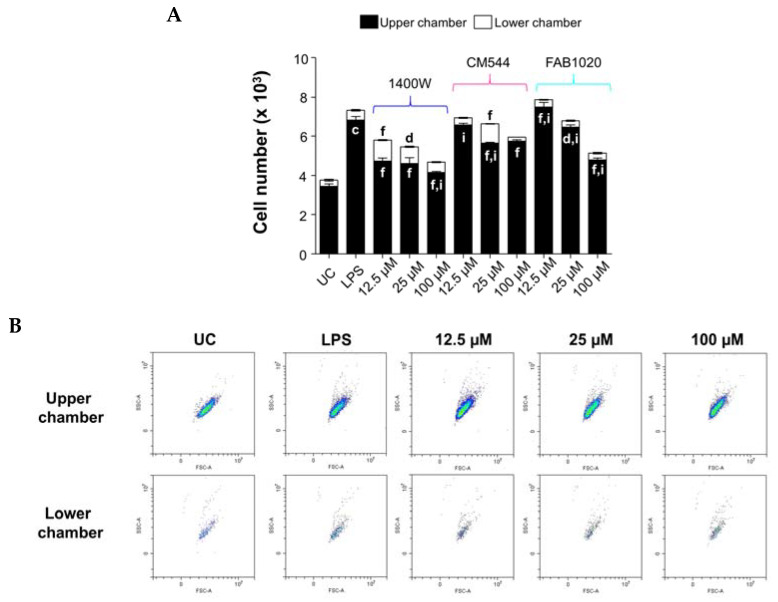
Cell migration in LPS-stimulated human monocytes in the presence of increasing concentrations of 1400 W, CM544 and FAB1020 after 24 h. (**A**) The bar graph represents the number of cells in the upper and the lower part of a Boyden chamber used for the migration assay. c = *p* < 0.0001 between LPS-stimulated cells and untreated cells. d = *p* < 0.01 and f = *p* < 0.0001 between LPS-stimulated cells and LPS-stimulated cells treated with compounds; i = *p* < 0.0001 between LPS-stimulated cells treated with 1400 W and LPS-stimulated cells treated with CM544 and FAB1020 at the same concentration. (**B**) Representative morphological dot plot (SSC/FSC) obtained by flow cytometry during the cell count of human monocytes in the Boyden chamber in the presence of FAB1020.

Migration is a fundamental early immune response in which activated polarized monocytes leave the circulation through permeable venular walls, differentiate into macrophages, and migrate towards inflammatory sites. Inhibiting the deleterious progression that is usually induced by macrophage infiltration is a challenge in many diseases [28]. Moreover, the expression of iNOS has been commonly used as a signature marker for monocyte pro-inflammatory polarization and migration [29]. In our experimental model, both iNOS inhibitors CM544 and FAB1020 decrease the number of migrating cells with respect to LPS alone after 24 h (Figure 5A).

In more detail, 0.25 µg/mL LPS enhances migration (0.5 × 10^3^ cells) with respect to the untreated control (0.305 × 10^3^ cells). In the presence of 100 µM CM544, the number of migrated monocytes is even lower (0.205 × 10^3^ cells) than that of untreated cultures. As for FAB1020, the inhibition of migration is remarkable already at 12.5 µM (0.365 × 10^3^ cells), and is maintained up to 100 µM (Figure 5B). In parallel, the total number of cells decreases in a dose-dependent manner in the presence of CM544 and even more so with FAB1020 with respect to LPS alone, confirming the cell metabolic activity data. Although the presence of loading concentrations of 1400 W are effective in decreasing the total number of cells as shown also by the MTS assay, this compound is not as efficient in lowering migration. As a matter of fact, the number of migrated monocytes is assessed at 1.06 × 10^3^ cells (12.5 µM), 0.832 × 10^3^ cells (25 µM) and 0.531 × 10^3^ cells (100 µM).

### 2.6. Modulation of iNOS and Protein Nitrosylation

After having evaluated the biological effect of the three iNOS inhibitors in terms of cytotoxicity, metabolic activity, migration and IL-6 secretion, a molecular analysis was performed. Excessive NO production from the iNOS is associated with the formation of peroxynitrite (ONOO^−^), as a reaction product of nitric oxide and superoxide radicals. ONOO^−^ interacts with cell membrane phospholipids and proteins, causing damage. S-nitrosylation of proteins can be assessed by the formation of nitrotyrosine in monocytes under inflammatory conditions [30].

**Figure 6:**
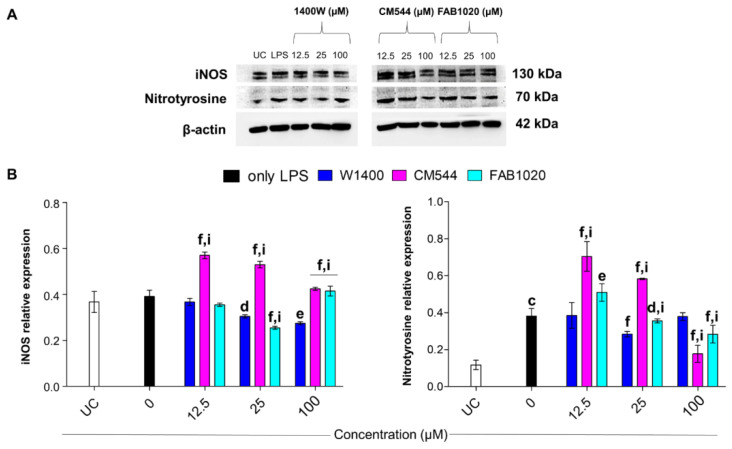
iNOS and nitrotyrosine protein expression in LPS-stimulated human monocytes in the presence of increasing concentrations of 1400 W, CM544 and FAB1020 after 24 h. (**A**) Protein bands from a representative experiment. β-actin is used as a loading control. (**B**) Bar graphs display densitometric values expressed as mean densitometric optical intensities (D.O.I) normalized on those of the loading control. UC = untreated cells. c = *p* < 0.0001 between LPS-stimulated cells and untreated cells. d = *p* < 0.01, e = *p* < 0.001 and f = *p* < 0.0001 between LPS-stimulated cells and LPS-stimulated cells treated with compounds; i = *p* < 0.0001 between LPS-stimulated cells treated with 1400 W and LPS-stimulated cells treated with CM544 and FAB1020 at the same concentration.

Although the iNOS expression is sightly induced by LPS after 24 h, the amount of nitrotyrosine is remarkable with respect to untreated cultures (Figure 6A,B). As reported previously, iNOS is expressed at low levels in human peripheral blood monocytes [24]. It has been broadly reported that LPS induces ONOO^−^ formation [31], and it is thus plausible to assume that the LPS-induced oxidative and nitrosative stress rapidly occurs, leading to early protein nitrosylation. Although iNOS expression is significantly decreased in the presence of loading concentration of 1400 W with respect to LPS alone, the compound is only slightly effective in lowering nitrotyrosine levels even at 100 µM. On the contrary, CM544 paradoxically induces iNOS expression in LPS-stimulated cells but is the most effective compound in lowering protein nitrosylation at 100 µM. As regards FAB1020, it is able to inhibit iNOS expression up to 25 µM, whereas it lowers nitrotyrosine levels in a dose-dependent manner, being more effective than 1400 W at 100 µM.

In monocytes, cell responses towards LPS-induced inflammatory responses are tightly modulated by the formation of ROS and RNS, which are the result of a finely regulated balance between the NO-NfΚB-NOX2-iNOS axis and the H_2_O_2_-Nrf2-HO-1 axis [27]. In this light, the expression of Nrf2 was therefore evaluated.

### 2.7. Expression of Nrf-2

**Figure 7:**
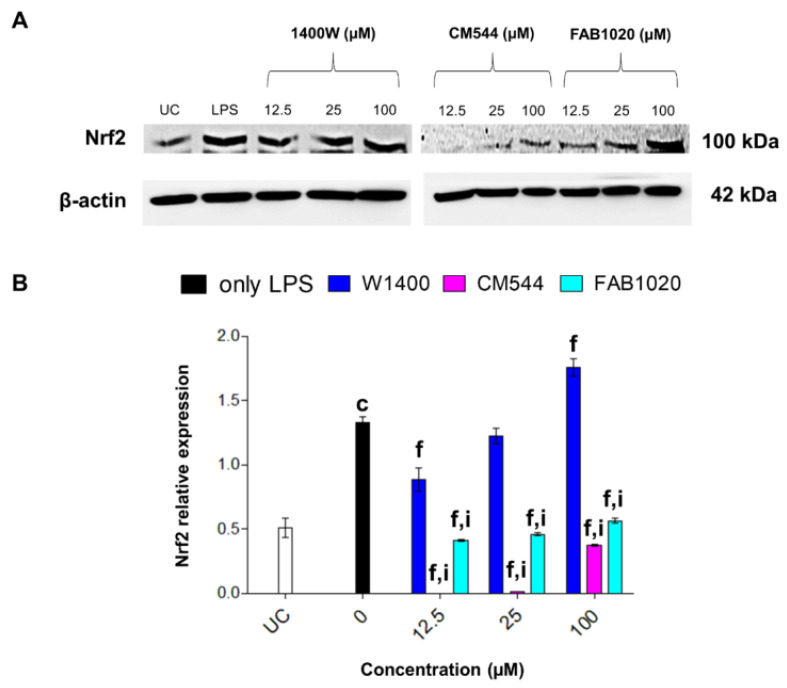
Nrf2 protein expression in LPS-stimulated human monocytes in the presence of increasing concentrations of 1400 W, CM544 and FAB1020 after 24 h. (**A**) Protein bands from a representative experiment. β-actin is used as loading control. (**B**) Bar graphs display densitometric values expressed as mean densitometric optical intensities (D.O.I) normalized on those of the loading control. UC = untreated cells. c = *p* < 0.0001 between LPS-stimulated cells and untreated cells. f = *p* < 0.0001 between LPS-stimulated cells and LPS-stimulated cells treated with compounds; i = *p* < 0.0001 between LPS-stimulated cells treated with 1400 W and LPS-stimulated cells treated with CM544 and FAB1020 at the same concentration.

As expected, Nrf2 is dramatically increased in LPS-stimulated monocytes with respect to untreated cultures, as a sign of nitrosative stress occurrence (Figure 7A,B). Increasing concentrations of 1400 W reduce Nrf2 levels up to 25 µM, but then the protein is overexpressed at the highest concentration administered. In the presence of CM544, Nrf2 is hardly detectable, except for the 100 µM concentration. In parallel, increasing concentrations of FAB1020 clearly inhibit Nrf2 expression in LPS-induced monocytes.

## 3. Materials and Methods

### 3.1. Synthesis of iNOS Inhibitors

The two selective iNOS inhibitors, CM544 and FAB1020, were synthesized, purified, and characterized as previously reported [14,16,32]. 1400 W was purchased from Merck (Darmstadt, Germany). Compounds were stored at −20 °C.

### 3.2. Cell Cultures

Human monocytes (CRL-9855™) were purchased from ATCC^®^ and sub-cultured in RPMI 1640 (EuroClone, Milan, Italy) supplemented with 10% heat-inactivated fetal bovine serum (FBS), 1% penicillin/streptomycin and 1% sodium pyruvate (all from Gibco, Invitrogen, Life Technologies, Carlsbad, CA, USA) at 37 °C and 5% CO_2_.

Primary human dental pulp cells (HDPCs) were obtained as previously reported [33]. According to the Italian Legislation and the code of ethical principles for medical research involving human subjects of the World Medical Association (Declaration of Helsinki), young donors who underwent extraction of the third molar signed an informed consent form. This project has received the approval of the Local Ethical Committee of the University of Chieti-Pescara (approval number 1173, date of approval 31 March 2016). HPCs were maintained in MEM alpha (EuroClone, Milan, Italy) supplemented with 10% heat-inactivated fetal bovine serum (FBS) and 1% penicillin/streptomycin (all from Gibco, Invitrogen, Life Technologies, Carlsbad, CA, USA).

### 3.3. Cell Metabolic Activity (MTS Test)

The MTS test was performed in 96-well plates (Thermo Fisher Scientific, Waltham, MA, USA) as a measure of cell metabolic activity. In a first set of experiments, human monocytes (0.5 × 10^5^ cells/well) and HDPCs (0.7 × 10^5^ cells/well) were exposed to loading concentrations of 1400 W, CM544 and FAB1020 (0–400 µM). In a second set of experiments, loading concentrations of all compounds (0–200 µM) were administered on human monocytes in the presence of lipopolysaccharide (LPS) 0.25 µg/mL. The concentration of LPS (from *E. coli*, purchased form Merck, Darmstadt, Germany) was chosen in accordance with preliminary results (Figure S1). At the established time point (24 h), the incubation medium was harvested for further analyses and complete RPMI containing 3-(4,5-dimethylthiazol-2-yl)-5-(3-carboxymethoxyphenyl)-2-(4-sulfophenyl)-2H-tetrazolium (MTS) at a concentration of 0.5 mg/mL was added to each well. Cells were incubated for 4 h at 37 °C and 5% CO_2_. After that, absorbance was measured at 490 nm using a spectrophotometer (Multiscan GO, Thermo Fisher Scientific, Waltham, MA, USA). The percentage of metabolically active cells in treated cultures was calculated, setting the untreated control to 100%.

### 3.4. Cytotoxicity Assay

The release of LDH into cell supernatants was quantified by the CytoTox 96^®^ nonradioactive assay (Promega Corporation, Fitchburg, WI, USA) to assess cytotoxicity in monocytes after a 24 h exposure period. Cell supernatants analyzed were collected from cultures analyzed by the MTS assay. The assay was carried out as previously reported [34]. LDH released from treated cell cultures was normalized to MTS absorbances and expressed as fold increases compared to untreated cultures set to 1.

### 3.5. Interleukin-6 (IL-6) Release

Amounts of IL-6 (pg/mL) were quantified in cell culture supernatants after a 24 h exposure period using a commercial ELISA kit (Enzo Life Sciences Inc, Lausen, Switzerland) as reported previously [35]. Cytokine concentrations were calculated from standard curves using the Prism5 software (GraphPad, San Diego, CA, USA), and these values were normalized to MTS data.

### 3.6. Migration Assay

The quantitative migration assay was performed using a modified Boyden chamber (Transwell^®^, Corning, NY, USA) with a pore polycarbonate filter (pore size = 8.0 µm) inserted in a 24-well plate. Untreated monocytes (0.5 mL) of a starved cell suspension were seeded into the upper chamber (2.5 × 10^3^) in different culture conditions: untreated cells, LPS 0.25 µg/mL and 1400 W, CM544 or FAB1020 at 12.5. 25 and 100 µM. The lower chamber was filled with 0.6 mL of complete RPMI 1640 medium. Following a 24 h incubation period, cells were harvested separately from the upper and the lower chamber and the number of cells in each compartment was assessed by flow cytometry (CytoFLEX, Beckman Coulter, CA, USA). Before running samples, morphological parameters (side scatter/forward scatter, SSC/FSC) and a defined acquisition time (1 min) were set.

### 3.7. Protein Extraction and Immunoblotting

After treating cells (2.5 × 10^4^/mL) in 6-well plates (ThermoFisher Scientific, Waltham, MA USA) for 24 h as previously described for the MTS assay, cells were collected by centrifugation (1000 rpm) in the cold and washed in cold PBS. Cell pellets were lysed and the amounts of proteins were quantified by a bicinchoninic acid (BCA) assay (Merck, Darmstadt, Germany), as reported elsewhere [36]. After lysis and quantification, 20 μg of the proteins of each sample were separated by 4–20% SDS-PAGE (ExpressPlus. 10 × 8, GenScript Biotech Corporation, Nanjing, China). After that, proteins were transferred to nitrocellulose membranes as described [33]. Next, membranes were blocked in 5% nonfat milk PBST (PBS plus 0.1% Tween 20, pH 7.4) and incubated in the presence of mouse monoclonal anti-β-actin (1:10,000) (Sigma-Aldrich, St. Louis, MO, USA) rabbit monoclonal anti-iNOS (1:200), mouse monoclonal anti-nitrotyrosine (1:200), or rabbit polyclonal anti-Nrf2 (1:750) (Santa Cruz Biotechnology, Santa Cruz, CA, USA). After an overnight incubation at 4 °C with primary antibodies under gentle shaking, membranes were then probed with specific IgG horseradish peroxidase (HRP)-conjugated secondary antibodies and bands were identified by chemiluminescence as previously described [36]. At least three independent experiments were performed for each protein. Results are expressed as mean values ± standard deviations (S.D.) of normalized densitometric values on the loading control (β-actin).

### 3.8. Statistical Analysis

Statistics were performed using one-way analysis of variance (ANOVA) followed by Tukey’s multiple comparison test by means of the Prism 5.0 software (GraphPad, San Diego, CA, USA). Results are shown as mean values ± standard deviations. Values of *p* ≤ 0.05 were considered statistically significant.

## 4. Conclusions

Nitric oxide is highly involved in monocyte functions, migration and differentiation, and modulating its bioavailability in these cells could affect their response to pro-inflammatory stimuli. In this study, the effects of CM544 and FAB1020, two potent and selective iNOS inhibitors, were investigated in LPS-stimulated human monocytes with regard to their capability to affect cell proliferation, migration, and inflammatory cell responses in terms of IL-6 secretion in comparison with the broadly known iNOS inhibitor 1400 W.

Very interestingly, these compounds were selective on the human monocyte cell line, without affecting human dental pulp cell metabolic activity. Furthermore, both CM544 and FAB1020 were able to modulate LPS-treated monocytes activation and proliferation and to decrease LPS-induced cytotoxicity and migration. Surprisingly, an over expression of iNOS occurs. Nevertheless, the augmented iNOS expression-levels are paralleled by the decrease in protein nitrosylation in the presence of FAB1020 compared to CM544 and 1400 W. Remarkably, CM544 and FAB1020, but not 1400 W, were able to lower Nrf2 and the pro-inflammatory cytokine IL-6, as a sign of cell response towards LPS-induced inflammation.

Taken together, these results highlight the biological effectiveness of CM544 and FAB1020, and therefore expand the knowledge of the immunomodulatory effects that proper iNOS inhibition can exert in monocytes. Our data demonstrate the potential outcomes in the research of new therapeutic strategies to target pathological conditions where an imbalanced immune response occurs.

## Data Availability

No data are reported.

## Supplementary Materials

The following are available online, Figure S1: Metabolic activity in the presence of increasing concentrations of LPS in CRL 9855 human monocytes and HDPCs after 24 and 72 h. The bar graphs represent the percentage of metabolically active cells in the presence of LPS (0–20 µg/mL). The control is represented by untreated cultures and is set as 100%. a = *p* < 0.01, b = *p* < 0.001 and c = *p* < 0.0001 between LPS-treated cells and the untreated control. Figure S2: Schematic drawing of the most important interactions of CM544 into the iNOS catalytic site. The CM544 acetamidine moiety interacts by a bidentate hydrogen bond (dotted lines) with a GLU and a TRP residues of the iNOS binding site. The aromatic ring is positioned atop one of the enzyme haeme pyrrole rings. The haeme propionate arms interact with the amide N-H group and the proline amino group by means of further hydrogen bonds.

## Sample Availability

Samples of the compounds CM544 and FAB1020 are available from the authors.
