# Supplementary material for: Selective Inhibitors of the Inducible Nitric Oxide Synthase as Modulators of Cell Responses in LPS-Stimulated Human Monocytes
Source: Molecules. 2021 Jul 22;26(15):4419. doi: 10.3390/molecules26154419 (PMC8348305; doi:10.3390/molecules26154419)
Supplement: Supplementary file 1 [file molecules-26-04419-s001.zip › molecules-1275507-supplementary.pdf]

Selective inhibitors of the inducible Nitric Oxide Synthase as modulators of cell responses in LPS-stimulated human monocytes

Marialucia Gallorini[1], Monica Rapino[2], Helmut Schweikl[3], Amelia Cataldi[1], Rosa Amoroso[1]*, Cristina Maccallini[1]

1 Department of Pharmacy, University "G. d'Annunzio" Chieti-Pescara, Via dei Vestini 31, 66100 Chieti, Italy;

2 Genetic Molecular Institute of CNR, Unit of Chieti, "G. d' Annunzio" University, Via dei Vestini 31, 66100, Chieti-Pescara, Italy

3 Department of Conservative Dentistry and Periodontology, University Hospital Regensburg, D-93042 Re-gensburg, Germany

* Correspondence: rosa.amoroso@unich.it

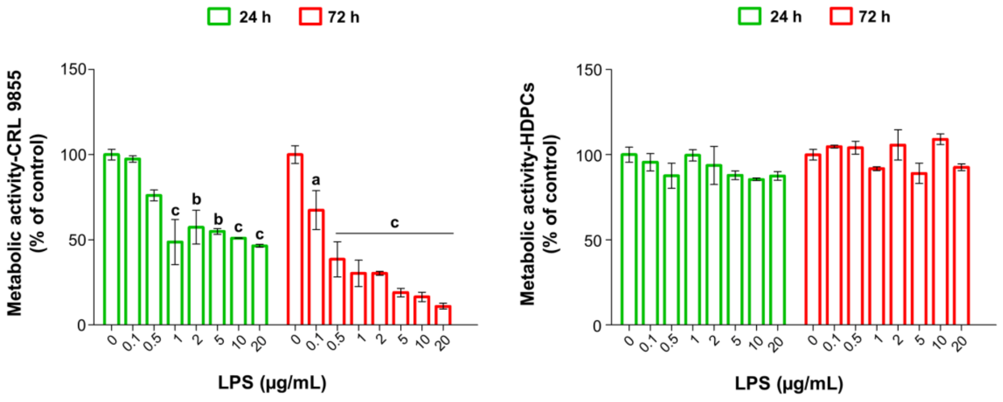

Figure S1. Metabolic activity in the presence of increasing concentrations of LPS in CRL 9855 human monocytes and HDPCs after 24 and 72 h. The bar graphs represent the percentage of metabolically active cells in the presence of LPS (0-20 μg/mL). The control is represented by untreated cultures and is set as 100%. a = $p<0.01$, b = $p<0.001$ and c = $p<0.0001$ between LPS-treated cells and the untreated control.

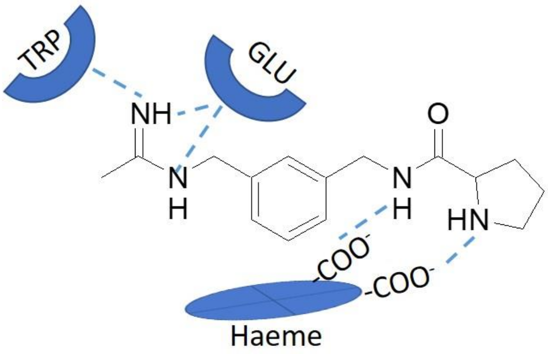

Figure S2. Schematic drawing of the most important interactions of CM544 into the iNOS catalytic site. The CM544 acetamidine moiety interacts by a bidentate hydrogen bond (dotted lines) with a GLU and a TRP residues of the iNOS binding site. The aromatic ring is positioned atop one of the enzyme haeme pyrrole rings. The haeme propionate arms interact with the amide N-H group and the proline amino group by means of further hydrogen bonds.
